# Anti-SARS-CoV2 antibody-mediated cytokine release syndrome in a patient with acute promyelocytic leukemia

**DOI:** 10.1186/s12879-022-07513-0

**Published:** 2022-06-13

**Authors:** Ahmed N. Hegazy, Jan Krönke, Stefan Angermair, Stefan Schwartz, Carl Weidinger, Ulrich Keller, Sascha Treskatsch, Britta Siegmund, Thomas Schneider

**Affiliations:** 1 Department of Gastroenterology, Infectious Diseases and Rheumatology, Charité-Universitätsmedizin Berlin, Corporate Member of Freie Universität and Humboldt Universität zu Berlin, Campus Benjamin Franklin, Berlin, Germany; 2 Deutsches Rheumaforschungszentrum Berlin (DRFZ), An Institute of the Leibniz Association, Berlin, Germany; 3 Berlin Institute of Health (BIH), Berlin, Germany; 4 Department of Hematology, Oncology and Tumor Immunology, Charité-Universitätsmedizin Berlin, Corporate Member of Freie Universität Berlin and Humboldt-Universität zu Berlin, Berlin, Germany; 5 Department of Anesthesiology and Intensive Care Medicine, Charité-Universitätsmedizin Berlin, Corporate Member of Freie Universität and Humboldt Universität zu Berlin, Campus Benjamin Franklin, Berlin, Germany

**Keywords:** Viral infection, Coronavirus disease 2019, SARS-CoV2, Antibody-dependent enhancement, Cytokine release syndrome, Acute promyelocytic leukemia, Case report

## Abstract

**Background:**

Passive immunization against SARS-CoV-2 limits viral burden and death from COVID-19; however, it poses a theoretical risk of disease exacerbation through antibody-dependent enhancement (ADE). ADE after anti-SARS-CoV2 antibody treatment has not been reported, and therefore the potential risk and promoting factors remain unknown.

**Case presentation:**

A 75-year-old female was admitted to the emergency room with recurrent, unexplained bruises and leukocytopenia, anemia, and thrombocytopenia. Evaluation of a bone marrow biopsy established the diagnosis of an acute promyelocytic leukemia (APL). SARS-CoV-2 RT-PCR testing of nasal and throat swabs on admission was negative. During the routine SARS-CoV-2 testing of inpatients, our patient tested positive for SARS-CoV-2 on day 14 after admission without typical COVID-19 symptoms. Due to disease- and therapy-related immunosuppression and advanced age conferring a high risk of progressing to severe COVID-19**,** casirivimab and imdevimab were administered as a preemptive approach. The patient developed immune activation and cytokine release syndrome (CRS) occurring within four hours of preemptive anti-SARS-CoV2 antibody (casirivimab/imdevimab) infusion. Immune activation and CRS were evidenced by a rapid increase in serum cytokines (IL-6, TNFα, IL-8, IL-10), acute respiratory insufficiency, and progressive acute respiratory distress syndrome.

**Discussion and conclusion:**

The temporal relationship between therapeutic antibody administration and the rapid laboratory, radiological, and clinical deterioration suggests that CRS was an antibody-related adverse event, potentially exacerbated by APL treatment-mediated differentiation of leukemic blasts and promyelocytes. This case highlights the need for careful assessment of life-threatening adverse events after passive SARS-CoV-2 immunization, especially in the clinical context of patients with complex immune and hematological landscapes.

**Supplementary Information:**

The online version contains supplementary material available at 10.1186/s12879-022-07513-0.

## Background

The profound global health, social, economic disruption from COVID-19 continues [1]. Despite the success of active and passive immunization strategies, antibody-based therapeutics pose a risk of exacerbating COVID-19 through antibody-dependent enhancement (ADE) and consequent enhanced virus replication or cytokine release syndrome (CRS) [2, 3]. Various antibody-based vaccines and therapeutics can promote ADE [2, 4–6], and while the extent to which ADE contributes to COVID-19 immunopathology is still being evaluated, the possible clinical risks related to anti-SARS-CoV-2 antibody treatment remain unclear. Anti-SARS-CoV-2 antibodies casirivimab and imdevimab have been authorized by the FDA for emergency use for patients with confirmed SARS-CoV-2 infection at high risk of severe COVID-19 and/or hospitalization, including immunosuppressed patients [7–9]. Until now, contraindications, which might preclude the use of these antibodies are undefined. Thus, meticulous clinical assessment of possible adverse events might help to further define their clinical application, and could guide decision-making [8, 10].

## Case presentation

**Fig. 1:**
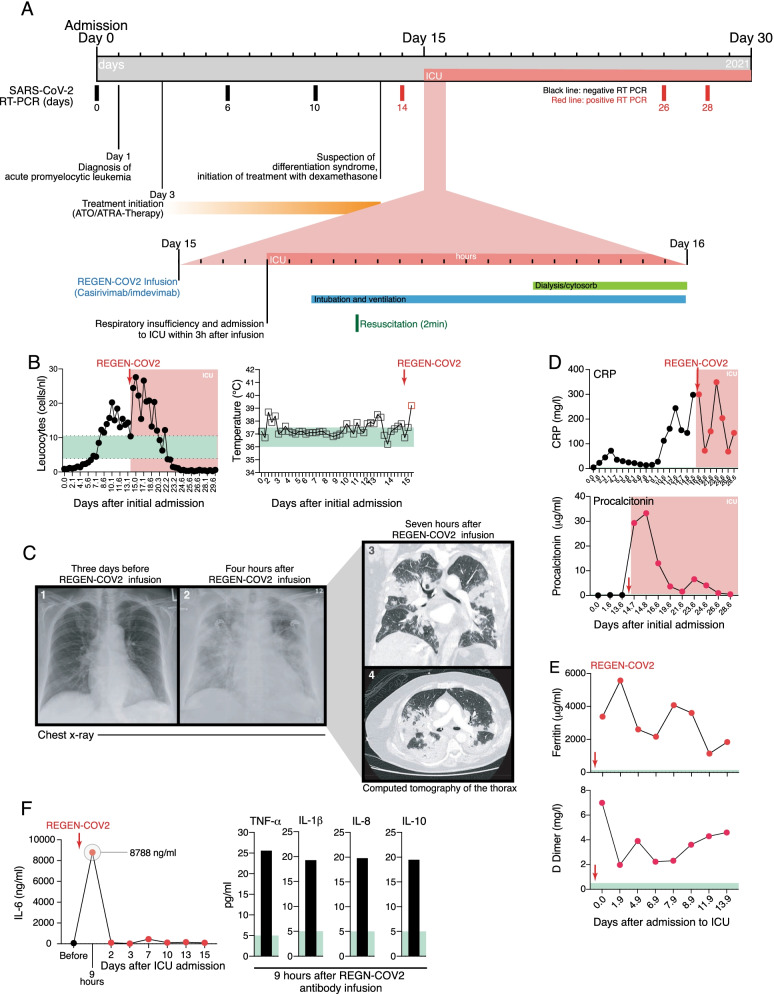
Clinical course, laboratory and radiological findings. (**A**) Clinical timeline from diagnosis of acute promyelocytic leukemia (APL) to CRS after casirivimab/imdevimab treatment. Diagram includes initiated treatments, SARS-CoV-2 PCR results and ICU admission and subsequent treatments. (**B**) Leucocyte count and body temperature. (**C**) Chest radiographs and subsequent CT scan of the chest. (**D**) CRP and procalcitonin during the course of admission. Red dots represent measurements after casirivimab/imdevimab and ICU admission. (**E**) Ferritin and D-Dimer levels after casirivimab/imdevimab treatment. Red arrow (REGEN-COV2) indicates the infusion of casirivimab/imdevimab. (**F**) Serum IL-6, TNF-α, IL-1β, IL-8 and IL-10 levels before and after casirivimab/imdevimab treatment. Red dots represent measurements after casirivimab/imdevimab infusion and ICU admission. Normal ranges are highlighted in green

A 75-year-old female was admitted to the emergency room with recurrent, unexplained bruises and leukocytopenia, anemia, and thrombocytopenia in mid-2021 (Fig. 1A, B and Additional file 1: Fig. S1A–D). Plasma coagulation was normal (INR 1.25, normal 0.9–1.25; aPTT 29.1, normal 25–38 s). She was referred to the hematology-oncology department for further assessment and diagnosis. Evaluation of a bone marrow biopsy established the diagnosis of an acute promyelocytic leukemia (APL) with characteristic Faggot cells and abnormal promyelocytes in the blood and bone marrow (Additional file 1: Fig. S1B). Cytogenetic and PCR analysis confirmed presence of a disease-defining, chromosomal 15;17-translocation. All-trans retinoic acid (ATRA) was initiated, and, subsequently, arsenic trioxide (ATO) was combined as part of the standard treatment for low-risk APL (Additional file 1: Fig. S1A) [11].

Patients with APL can develop differentiation syndrome (DS), a potentially fatal complication of treatment with differentiation-inducing agents, such as ATRA and ATO, through promoting the differentiation of leukemic cells (i.e. atypical promyelocytes) [12]. Therefore, 30 mg/kg/day oral prednisolone was started as prophylaxis (Fig. 1A and Additional file 1: Fig. S1A) [11]. Due to pyrexia of 38.8 °C on day 2 of admission (Fig. 1B and Additional file 1: Fig. S1E), blood and urine cultures and chest X-ray were performed to exclude infection (Fig. 1C, Additional file 1: Fig.S 2 and Additional file 1: Table S1). SARS-CoV-2 RT-PCR testing of nasal and throat swabs on admission was negative (Fig. 1A and Additional file 1: Table S2), and cytomegalovirus (CMV) and Epstein–Barr virus (EBV) PCR tests on blood samples were also negative (Additional file 1: Table S2). Empiric antibiotic therapy with piperacillin/tazobactam was initiated due to her immunosuppression (Additional file 1: Fig. S1A).

After ten days of ATRA/ATO, the patient’s CRP increased but she remained subfebrile (37.5–38 °C; Fig. 1B and D). Her blood counts showed increasing neutrophils, but unaltered lymphopenia and thrombocytopenia (Additional file 1: Fig. S1C). There were no chest X-ray changes from admission (Additional file 1: Fig. S2), repeat microbiological and SARS-CoV-2 testing remained negative, and procalcitonin levels were normal (Fig. 1D, and Additional file 1: Table S2). Due to persistent unexplained fever and increased CRP in the absence of a documented infection, DS was suspected [12]. However, there was no respiratory distress, weight gain, pleural effusion, or pulmonary infiltrates, which are typical DS characteristics (Additional file 1: Fig. S2). Nevertheless, ATRO and ATO were stopped and hydroxycarbamide (2 g/day) was started on day 8 to prevent hyperleukocytosis together with dexamethasone (20 mg/day) to treat the suspected DS. Also, the empiric piperacillin/tazobactam was reinitiated after a brief pause. An ECG prompted by sustained tachycardia showed atrial fibrillation, thus bisoprolol was initiated on day 14 after admission.

During the routine SARS-CoV-2 testing of inpatients, our patient tested positive for SARS-CoV-2 on day 14 after admission (after the three previous negative tests on days 0, 6, and 10), but typical COVID-19 symptoms were absent (Fig. 1A and Additional file 1: Table S2). She was transferred to the infectious disease unit, where her vital signs were oxygen saturation 99% on room air; respiratory rate 24 breaths per minute; blood pressure 130/80 mmHg; heart rate 130 beats per minute; and temperature 37.8 °C. The CRP remained elevated and interleukin-6 (IL-6) was 60.1 ng/mL (normal < 7 ng/mL); procalcitonin 0.19 µg/mL (normal < 0.5 µg/ml); while the elevated neutrophil count, lymphocytopenia and thrombocytopenia persisted (Fig. 1D, E; Additional file 1: Figure S1C–E).

Due to disease- and therapy-related immunosuppression and advanced age conferring a high risk of progressing to severe COVID-19, casirivimab and imdevimab were administered as a preemptive approach done according to the recommendation of emergency use authorization of REGEN-COV [7, 8]. Casirivimab and imdevimab (1200 mg each) were administered together as a single intravenous infusion over 90 min within 24 h of positive SARS-CoV-2 RT-PCR without any immediate adverse reactions. However, three hours after the infusion, the patient developed severe respiratory distress with an oxygen saturation dropping to 76% on room air; the respiratory rate increased to 42 breaths per minute; blood pressure 135/80 mmHg; heart rate 130 beats per minute; and temperature 39.2 °C (Fig. 1B and Additional file 1: Fig. S1E). The respiratory insufficiency was not accompanied by any signs of a hypersensitivity reaction, and she was transferred to the intensive care unit where she received high-flow oxygen therapy. Inflammatory markers including ferritin and procalcitonin suddenly increased, and her D-dimers were also elevated (Fig. 1D, E). Due to the rapid clinical deterioration, antibody-mediated CRS was suspected and serum cytokines were evaluated. These were indeed elevated within six hours of casirivimab and imdevimab infusion, with IL-6 levels peaking at 8768 ng/mL (normal < 7 ng/mL) and elevated TNF-α, IL-1β, IL-8, and IL-10 (Fig. 1F). Within eight hours of ICU admission, she developed cardiac arrest and was resuscitated for two minutes until return of spontaneous circulation and intubated for mechanical ventilation (Fig. 1A and Additional file 1: Fig. S3A).

Cytokine storm is an umbrella term encompassing several disorders of immune dysregulation characterized by systemic inflammation and multiorgan dysfunction [13]. To differentiate pathogen-induced from antibody-mediated CRS, further laboratory and radiological assessments were performed. Chest radiographs revealed radiographic opacities and a subsequent CT scan showed severe atypical infiltrations resembling typical interstitial COVID-19 pneumonia, with no nidus of infection in the abdomen and pelvis or pulmonary artery embolism (Fig. 1C, and Additional file 1: Fig. S2). No antiviral therapy was initiated because of SARS-CoV2 infection because none were approved at the time. A full microbiological screen was performed (Additional file 1: Table S1) and empiric antibiotic and antifungal therapy with vancomycin, meropenem, and liposomal amphotericin B were initiated. However, all repeat microbiological tests over a two-week period during the ICU stay remained negative (Additional file 1: Table S1). Comprehensive microbiological analyses of bronchoscopy and bronchoalveolar lavage (BAL) samples were negative except for SARS-CoV-2 (Additional file 1: Tables S1 and S2). Having excluded systemic bacterial, fungal, and viral infections other than SARS-CoV-2, the diagnosis of antibody-mediated disease enhancement (ADE) and CRS became more likely, so continuous veno-venous hemodialysis with hemoadsorption (CytoSorb, CytoSorbents, USA) was commenced for 6.5 days together with intravenous 20 mg dexamethasone daily. After initial pronounced catecholamine requirement (norepinephrine 0.6 µg/kg/min), volume therapy, and several repetitions of the prone positioning therapy, the respiratory insufficiency improved significantly, so that the patient was extubated. Due to progressive hyperleukocytosis and thrombocytopenia as well as coexisting circulating promyelocytes in the blood, continuous cytarabine (100 mg/m^2^) was given for six days to treat a possible concurrent DS. After clinical stabilization and leucocyte decline, ATRA and ATO were reinitiated. Further assessment of the immune cell compartment during this treatment showed decreasing leukocytosis and granulocytosis, and the CD4/CD8 T cell ratio disclosed a relative increase in CD4^+^ T cells and decrease in CD8^+^ T cells (Additional file 1: Fig. S3B, C). The patient was discharged after 41 days from the ICU. At discharge, nasopharyngeal swabs for SARS-CoV-2 turned negative, her inflammatory markers had decreased (CRP 76.3 mg/L; LDH 229 U/L, PCT 0.21 µg/L) and a bone marrow examination confirmed complete morphologic remission of her APL.

## Discussion and conclusion

SARS-CoV-2 targets various cell types in multiple organs and induces an excessive inflammatory response promoting multi-organ damage [14]. The role of ADE in COVID-19 pathology has not been fully established and there is currently no definitive evidence demonstrating that ADE occurs with SARS-CoV-2 infection [2, 10, 15]. ADE can occur via two mechanisms. First, pathogen-specific antibodies could promote infection by enhancing virus uptake and replication in Fcγ receptor-expressing immune cells. ADE can alternatively be mediated via enhanced immune activation by Fc-mediated effector functions leading to cytokine release, disease exacerbation, and, in severe cases, acute respiratory distress syndrome [2]. Indeed, the Fc effector functions of casirivimab and imdevimab were left unaltered to promote therapy efficacy against SARS-CoV-2 [9]. In our case, DS-mediated accumulation of myeloid cells in different organs, especially the lung, could have led to overt inflammation, cytokine release, and ARDS through myeloid cell activation by immune complex formation upon infusion of casirivimab and imdevimab in the presence of high viral loads, most abundantly in the lungs [14]. Patients with APL can develop DS, a potentially fatal complication of treatment with differentiation-inducing agents such as ATRA and ATO [12]. ATRA and ATO act through the aberrant retinoid receptor PML-RARA to promote promyelocyte maturation resulting in an enhanced tissue infiltration. Post-mortem studies of patients with DS have revealed extensive myeloid cell infiltrates in various organs including the lung, lymph nodes, spleen, liver, and pericardium [16]. Its early diagnosis can be challenging due to non-specific signs and symptoms such as fever, myalgia, dyspnea, hypotension, peripheral and pulmonary edema, and pleural and pericardial effusions [12]. However, plasma cytokine levels remain relatively low, and their diagnostic value for DS in patients with APL is poor [17, 18].

Establishing the underlying cause of cytokine storm can be challenging, especially as the laboratory findings in CRS are variable and influenced by the underlying cause. The close temporal relationship between anti-SARS-CoV-2 antibody treatment and the development of CRS and ARDS in our case favors anti-SARS-CoV-2 antibody-mediated immune activation as the potential trigger. However, possible alternative triggers that could have promoted the above-mentioned clinical development include systemic bacterial infection and subsequent septic shock, allergic reaction to the different medications taken by the patient other than the anti-SARS-CoV2 antibodies, and APL-dependent hyperleukocytosis and differentiation syndrome mediated cytokine release syndrome. However, systemic bacterial infection was rather unlikely in our patient through repetitive microbiological assessment with negative findings (Additional file 1: Table S1). Furthermore, the rapid and extensive release of IL-6 (increasing 146-fold) within six hours of infusion and the absence of any signs of allergic or hypersensitivity reaction argues against allergic, primary COVID-19-related, or septic shock-induced cytokine release [19, 20].

This case report has its limitations that hinder us from having a definitive conclusion on the promoting factor of the observed CRS. First, this is a case study of one patient who developed CRS after anti-SARS-CoV antibody treatment with complicated underlying hematological disease, acute promyelocytic leukemia. Second, it is difficult to exclude other alternative triggers promoting the CRS, including bacterial infection, allergic reaction to anti-SARS-CoV2 antibodies or other medications, and APL-related DS. However, the unique immunological landscape of patients with hematological malignancies might be a key determinant of possible immune-related adverse events to monoclonal antibody therapies, especially the development of ADE-induced hyperinflammation and CRS. Given that patients with hematological disorders and cancer were excluded from trials evaluating SARS-CoV-2-specific neutralizing monoclonal antibodies, this case highlights the need for pharmacovigilance when using anti-SARS-CoV-2 antibodies in patients with cancer and immunological disorders. No ADE or CRS events have yet been reported after either bamlanivimab or casirivimab and imdevimab therapy. Current recommendations for the therapeutic use of anti-SARS-CoV-2 antibodies suggest administration to patients at risk, including those with immunocompromising diseases. However, new additional antiviral therapies have been introduced recently and provide additional options for patients with complex hematological diseases. While the benefit-risk profile for these anti-SARS-CoV-2 antibodies remains strongly in favor of their use, careful assessment of the underlying condition and possible immune activation upon antibody administration in the presence of high antigen load need careful consideration.

## Supplementary Information

**Additional file 1: Figure S1. ** (**A**) Clinical timeline highlighting the initiated hematological treatments of APL before SARS-CoV-2 detection in nasopharyngeal swabs. (**B**) Faggot cells with multiple Auer rods (arrow mark) in bone marrow aspirate. Blood differential tests (**C**), hemoglobin and LDH (**D**), and vital signs including blood pressure, heart rate, and oxygen saturation (**E**) from admission to casirivimab/imdevimab infusion. Normal ranges are highlighted in green. **Figure S2.** Chest X-rays along the clinical course from diagnosis of acute promyelocytic leukemia (APL) to CRS after casirivimab/imdevimab infusion and subsequent period in the ICU. **Figure S3.** (**A**) Clinical timeline highlighting the initiated treatments in the ICU after casirivimab/imdevimab infusion. (**B**) Complete blood count after ICU admission.  (**C**) Flow cytometric analysis of peripheral blood after ICU admission including CD3 T cell characterization. Normal ranges are highlighted in green. **Table S1.** Microbiological laboratory assessment from diagnosis of acute APL to CRS after casirivimab/imdevimab treatment and during the ICU stay. (**A**) Microbiological culture performed on different patient-derived materials. (**B**) Microbiological assays for the analysis of certain pathogens. **Table S2.** Virological laboratory assessment from diagnosis of acute APL to CRS after casirivimab/imdevimab treatment and during the ICU stay. (**A**) SARS-CoV-2 molecular diagnostics including RT-PCR and virus sequencing at different time points. (**B**) Serological assessment of the immune status to different viruses. (**C**) PCR detection of various viruses in blood and bronchoalveolar lavage.

## Data Availability

All laboratory tests, included in the case report, were performed in the laboratories of Labor Berlin GmbH (Berlin), following the standard procedures. The datasets generated and analysed during the current study are available from the corresponding author on reasonable request.

## Abbreviations

ADE: Antibody-dependent enhancement
APL: Acute promyelocytic leukemia
ATO: Arsenic trioxide
ATRA: All-trans retinoic acid
CMV: Cytomegalovirus
CRS: Cytokine release syndrome
EBV: Epstein–Barr virus
ICU: Intensive care unit
IL: Interleukin
LDH: Lactate dehydrogenase
RT-PCR: Reverse transcription-polymerase chain reaction
RSV: Respiratory Syncytial Virus
TNFα: Tumor necrosis factor-alpha
