# Supplementary material for: Anti-SARS-CoV2 antibody-mediated cytokine release syndrome in a patient with acute promyelocytic leukemia
Source: BMC Infect Dis. 2022 Jun 13;22:537. doi: 10.1186/s12879-022-07513-0 (PMC9188919; doi:10.1186/s12879-022-07513-0)
Supplement: Supplementary file 1 — Additional file 1: Figure S1. (A) Clinical timeline highlighting the initiated hematological treatments of APL before SARS-CoV-2 detection in nasopharyngeal swabs. (B) Faggot cells with multiple Auer rods (arrow mark) in bone marrow aspirate. Blood differential tests (C), hemoglobin and LDH (D), and vital signs including blood pressure, heart rate, and oxygen saturation (E) from admission to casirivimab/imdevimab infusion. Normal ranges are highlighted in green. Figure S2. Chest X-rays along the clinical course from diagnosis of acute promyelocytic leukemia (APL) to CRS after casirivimab/imdevimab infusion and subsequent period in the ICU. Figure S3. (A) Clinical timeline highlighting the initiated treatments in the ICU after casirivimab/imdevimab infusion. (B) Complete blood count after ICU admission. (C) Flow cytometric analysis of peripheral blood after ICU admission including CD3 T cell characterization. Normal ranges are highlighted in green. Table S1. Microbiological laboratory assessment from diagnosis of acute APL to CRS after casirivimab/imdevimab treatment and during the ICU stay. (A) Microbiological culture performed on different patient-derived materials. (B) Microbiological assays for the analysis of certain pathogens. Table S2. Virological laboratory assessment from diagnosis of acute APL to CRS after casirivimab/imdevimab treatment and during the ICU stay. (A) SARS-CoV-2 molecular diagnostics including RT-PCR and virus sequencing at different time points. (B) Serological assessment of the immune status to different viruses. (C) PCR detection of various viruses in blood and bronchoalveolar lavage. [file 12879_2022_7513_MOESM1_ESM.pdf]

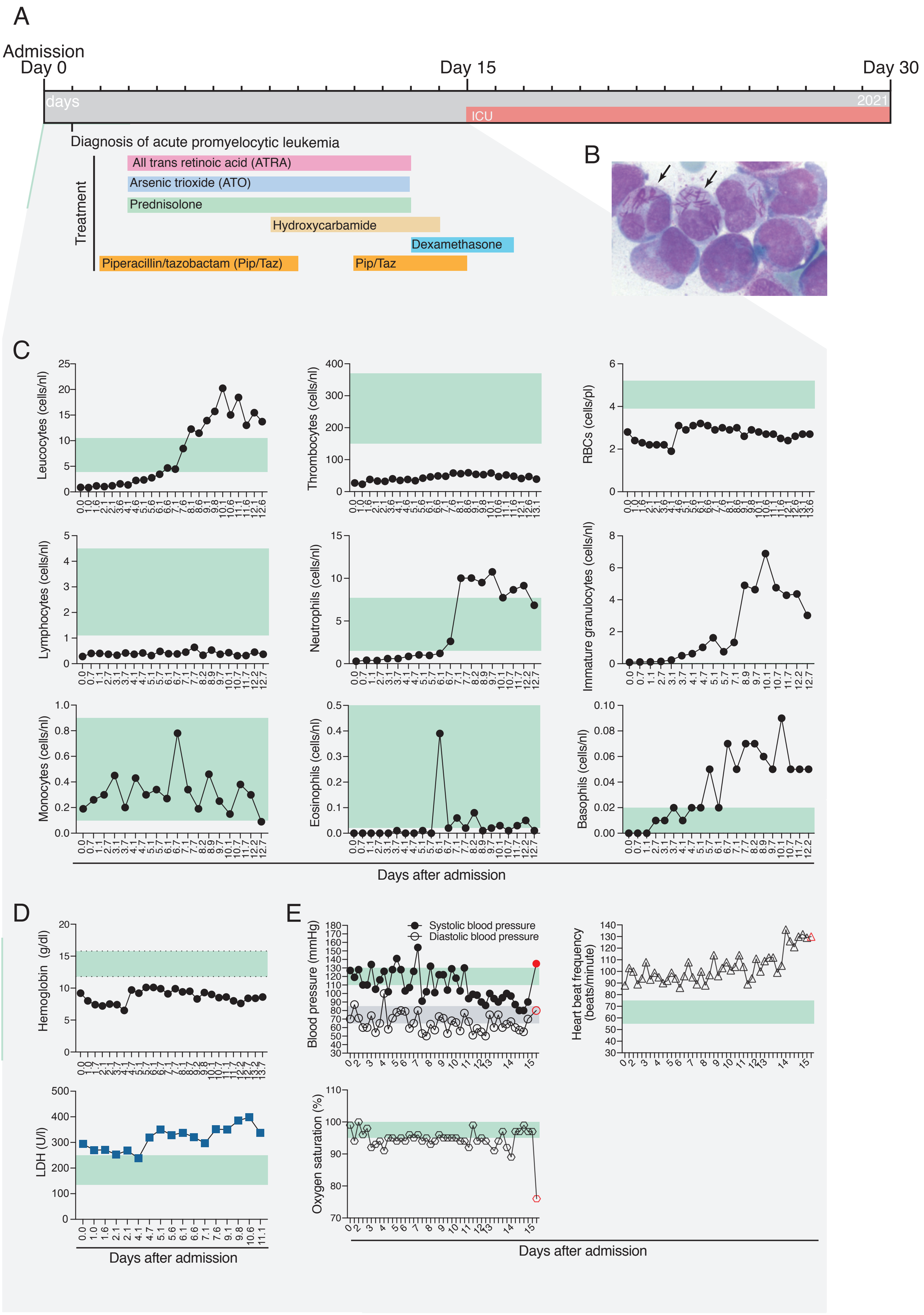

Supplementary figure 1

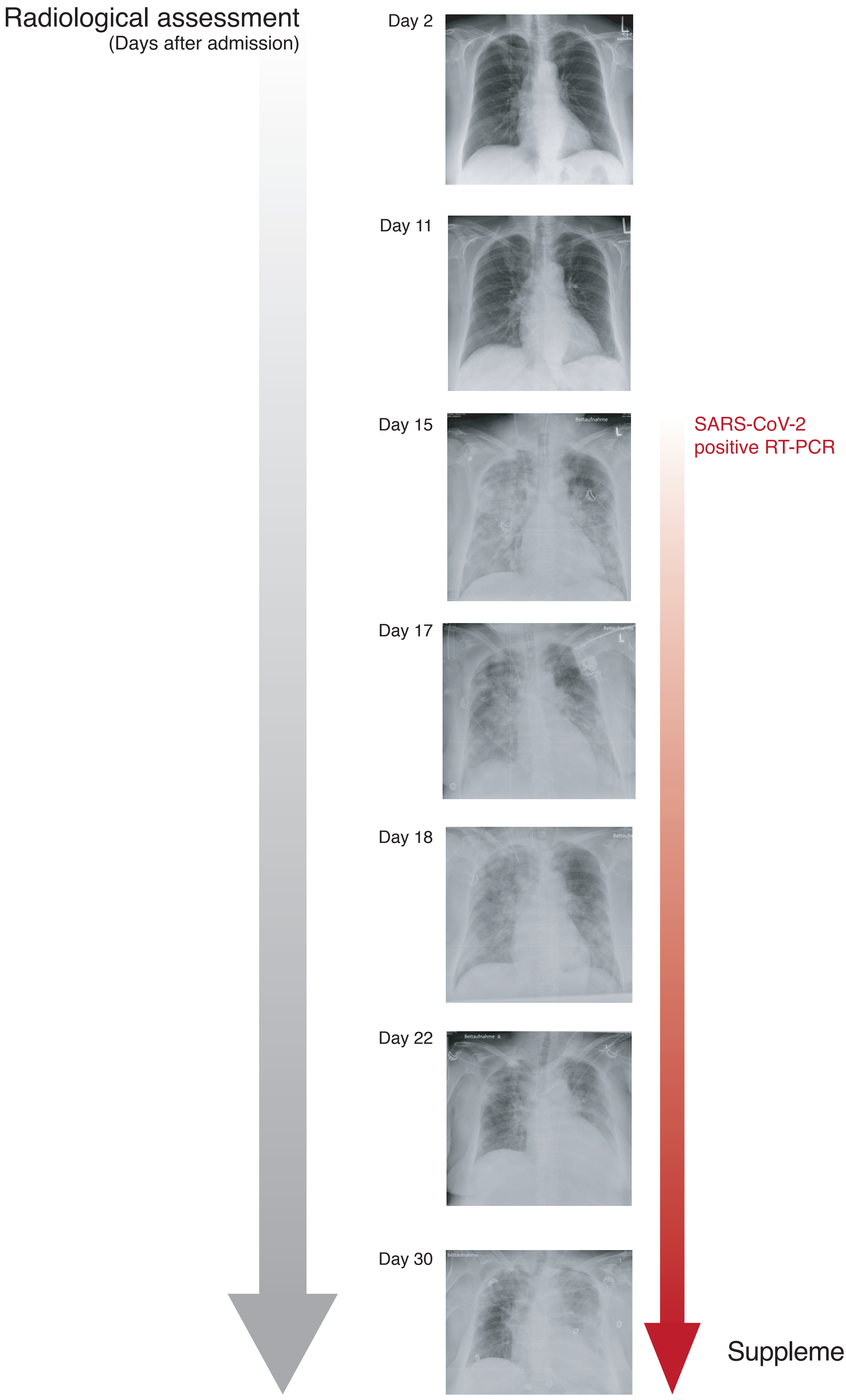

Supplementary figure 2

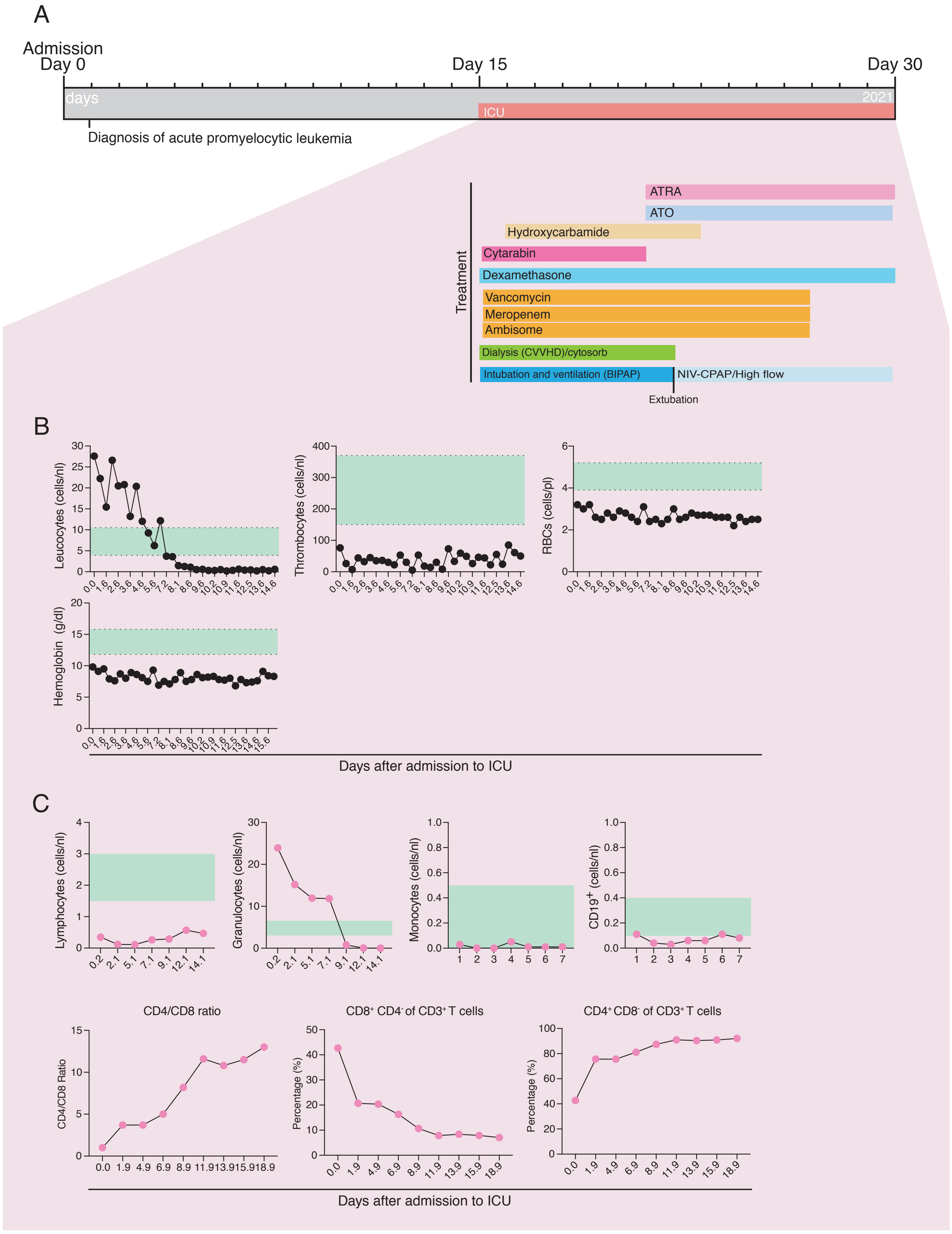

Supplementary figure 3

Supplementary Table S1

A. Microbiological cultures

**Days after admission**	**Tested material**	**Results**	**Comments**
Day 2	Blood culture	no bacterial growth	
Day 2	Blood culture	no bacterial growth	
Day 2	Urine culture	no bacterial growth	
Day 6	Stool	*Clostridium difficile* Glutamate Dehydrogenase - negative	
Day 9	Stool	*Clostridium difficile* Glutamate Dehydrogenase - negative	
Day 11	Blood culture	no bacterial growth	
Day 11	Urine culture	no bacterial growth	
Day 15	Blood culture	no bacterial growth	
Day 15	Blood culture	no bacterial growth	
Day 15	Blood culture	no bacterial growth	
Day 15	Blood culture	no bacterial growth	
Day 15	Blood culture	no fungal growth	
Day 15	Urine culture	no bacterial growth	
Day 18	Blood culture	no bacterial growth	
Day 18	Blood culture	no bacterial growth	
Day 18	Blood culture	no bacterial growth	
Day 24	Blood culture	no bacterial growth	
Day 24	Urine culture	no bacterial growth	
Day 24	Blood culture	no fungal growth	

B. Specific *microbiological assays*

**Days after admission**	**Tested material**	**Tested pathogen**	**Results**	**Comments**
Day 0	Serum	*Treponema pallidum*-specific antibodies	negative	
Day 15	BAL	*Pneumocystis jirovecii* antigen<br>*Pneumocystis jirovecii* IFT	negative<br>negative	BAL: Bronchoalveloar lavage
Day 17	Urine	*Leg. pneumophila*. Serotype 1-Antigen	negative	
Day 17	BAL	*Aspergillus* Antigen	negative	BAL: Bronchoalveloar lavage
Day 17	BAL	*Chlamydia pneumoniae*-DNA-PCR	negative	BAL: Bronchoalveloar lavage
Day 17	BAL	*Legionella pneumophila*-DNA-PCR	negative	BAL: Bronchoalveloar lavage
Day 17	BAL	*Mycoplasma pneumoniae-DNA-*PCR	negative	BAL: Bronchoalveloar lavage

Supplementary Table S2

A. SARS-CoV 2 molecular diagnostics

Days after admission	Tested material		Results	Comments
Day 0	nasopharyngeal swabs	SARS-CoV-2-RNA (PCR)	Not detected	
Day 6	nasopharyngeal swabs	SARS-CoV-2-RNA (PCR)	Not detected	
Day 10	nasopharyngeal swabs	SARS-CoV-2-RNA (PCR)	Not detected	
Day 14	nasopharyngeal swabs	SARS-CoV-2-RNA (PCR)	Positive	Ct Threshold: <30 cycles
Day 14	nasopharyngeal swabs	SARS-CoV-2 Sequencing	B.1.1.7 (17/17 B.1.1.7 SNPs)	Mutations in S-Gene:<br>- IHV68I: ND<br>- K417T: ND<br>- K417N: ND<br>- N439K: ND<br>- L452R: ND<br>- Y453F: ND<br>- E484K: ND<br>- E484Q: ND<br>- N501Y: D<br>- D614G: D<br>- Q677H: ND<br>- P681H: D<br>- P681R: ND<br>- V1176F: Not enough coverage<br><br>(ND: not detected; D: Detected)
Day 27	nasopharyngeal swabs	SARS-CoV-2-RNA (PCR)	Positive	Ct Threshold: <30 cycles
Day 29	nasopharyngeal swabs	SARS-CoV-2-RNA (PCR)	Positive	Ct Threshold: >30 cycles
Day 34	nasopharyngeal swabs	SARS-CoV-2-RNA (PCR)	Positive	Ct Threshold: >30 cycles

B. Viral serology

Days after admission	Tested material		Results	Comments
Day 1	Serum	HIV-DUO SE	negative	
Day 2	Serum	EBV-VCA-IgG (EIA)	>750 U/ml	
Day 2	Serum	EBV-VCA-IgM (EIA)	negative	
Day 2	Serum	EBV-EBNA1-IgG (EIA)	229 U/ml	
Day 2	Serum	CMV-IgG (EIA)	100.1 AE/ml	
Day 22	Serum	HIV-DUO SE	negative	
Day 23	Serum	HBc-Antibodies	negative	
Day 23	Serum	HBs-Antibodies	205 U/l	

Day 23	Serum	Hepatitis C Virus Antibodies	negative	
Day 23	Serum	HBs-Antigen	negative	

C. Molecular viral testing

Days after admission	Tested material		Results	Comments
Day 2	Blood	*Epstein-Barr Virus* (EBV) DNA - PCR	Not detected	
Day 2	Plasma	*Cytomegalovirus* (CMV)-DNA- PCR	Not detected	
Day 17	BAL	SARS-Coronavirus 2-RNA (Multiplex-PCR)	Positive	
Day 17	BAL	*Respiratory syncytial virus A*-RNA (Multiplex-PCR)	Not detected	
Day 17	BAL	*Respiratory syncytial virus B*-RNA (Multiplex-PCR)	Not detected	
Day 17	BAL	*Influenza A*-Virus-RNA (Multiplex-PCR)	Not detected	
Day 17	BAL	*Influenza B*-Virus-RNA (Multiplex-PCR)	Not detected	
Day 17	BAL	*Parainfluenza 1*-RNA (Multiplex-PCR)	Not detected	
Day 17	BAL	*Parainfluenza 2*-RNA (Multiplex-PCR)	Not detected	
Day 17	BAL	*Parainfluenza 3*-RNA (Multiplex-PCR)	Not detected	
Day 17	BAL	*Parainfluenza 4*-RNA (Multiplex-PCR)	Not detected	
Day 17	BAL	*Metapneumovirus*-RNA (Multiplex-PCR)	Not detected	
Day 17	BAL	*Coronavirus 229E*-RNA (Multiplex-PCR)	Not detected	
Day 17	BAL	*Coronavirus NL63*-RNA (Multiplex-PCR)	Not detected	
Day 17	BAL	*Coronavirus HKU1*-RNA (Multiplex-PCR)	Not detected	
Day 17	BAL	*Coronavirus OC43*-RNA (Multiplex-PCR)	Not detected	
Day 17	BAL	*Bocavirus*-DNA (Multiplex-PCR)	Not detected	
Day 17	BAL	*Influenza A H1N1/2009* (Multiplex-PCR)	Not detected	
Day 17	BAL	*Influenza A H1* (Multiplex-PCR)	Not detected	
Day 17	BAL	*Influenza A H3* (Multiplex-PCR)	Not detected	
Day 17	BAL	*Entero/Rhinovirus* (Multiplex-PCR)	Not detected	
Day 17	BAL	*Adenovirus*-DNA (Multiplex-PCR)	Not detected	
